# Real-world experience with ruxolitinib for steroid-refractory acute graft-versus-host disease: a single center experience

**DOI:** 10.1007/s12185-022-03434-5

**Published:** 2022-08-16

**Authors:** Adrianna Spałek, Agata Wieczorkiewicz-Kabut, Anna Koclęga, Krzysztof Woźniczka, Patryk Węglarz, Kinga Boral, Dariusz Kata, Patrycja Zielińska, Grzegorz Helbig

**Affiliations:** School of Medicine in Katowice, Department of Hematology and Bone Marrow Transplantation, Medical University of Silesia, Dąbrowski Street 25, 40-032 Katowice, Poland

**Keywords:** Acute graft-versus-host disease, Allogeneic hematopoietic stem cell transplantation, Corticosteroids, Refractoriness, Ruxolitinib

## Abstract

Steroid-refractory acute graft-versus-host disease (SR-aGVHD) remains a major cause of morbidity and mortality after allogeneic hematopoietic stem cell transplantation (allo-HSCT). Ruxolitinib (RUX), an oral JAK1 and JAK2 inhibitor, has recently been approved for patients with SR-aGVHD. The aim of this study was to evaluate RUX efficacy and toxicity in a real-world setting. Eighteen patients received RUX at 5 mg or 10 mg twice a day after a median 3 lines of prior unsuccessful immunosuppressive therapy. Median time on RUX therapy was 28 days (range 7–129). Five patients (28%) responded to RUX, including 4 complete responses and 1 partial response. Response to RUX was irrespective of aGVHD grade and the number of involved organs. One-year overall survival (OS) was 60% for RUX-responders versus 31% for non-responders (p = ns). Treatment duration greater than 29.5 days was found to have a positive impact on OS (*p* < 0.007). Major adverse events during RUX treatment were grade 3–4 thrombocytopenia (61% of patients) and cytomegalovirus reactivation (50%). After median follow-up of 55 days (range 29–706), 14 patients (78%) died, mainly due to further progression of GVHD. RUX may represent a valuable therapeutic option for some patients with advanced SR-aGVHD, but more studies are warranted.

## Introduction

Grades (G) III–IV acute graft-versus-host disease (aGVHD) remains a devastating complication after allogeneic hematopoietic stem cell transplantation (allo-HSCT) resulting in a high rate of mortality despite peri-transplant prophylaxis and early initiation of corticosteroids (CS). It has been over 60 years after the first reports of possible immunologic complications after murine bone marrow transplantation (1959) [1]. Since that time an effective therapy for advanced (GIII–IV) aGVHD still remains a challenge and only the use of high-doses CS is fully accepted as a first-line treatment. It has been estimated that nearly half of the graft recipients suffering from advanced aGVHD respond to initial therapy with CS [2]. Of note is, that lack of response to CS results in extremely short survival of approximately 40% at 6 months [3]. Nowadays, second-line treatment for steroid-refractory aGVHD (SR-aGVHD) differs between centers and depends on physician’s experience. The agents commonly used as second and further lines of therapy include anti-thymocyte globulin (ATG), mycophenolate mofetil, calcineurin inhibitors or extracorporeal photopheresis, however, the response varied [2].

A hope was supposed to come in 2019 after the Food and Drug Administration (FDA) approved Ruxolitinib (RUX), a JAK1/2 inhibitor, for therapy of SR-aGVHD in adult and pediatric patients > 12 years [4]. Approval was based on favourable results of REACH2 clinical trial in which RUX showed the advantage over other well-known immunosuppressive therapies. Promising response rate to RUX at day 28 was 62.3% when compared with 39.4% in control group (*p* < 0.001) [5].

Here, we report on our real-life data of RUX for advanced SR-aGVHD undergoing allo-HSCT in our center between years 2019 and 2021.

## Materials and methods

### Patients and methods

This retrospective study included 18 patients treated with RUX as a salvage therapy for GIII–IV SR-aGVHD. All transplanted patients received post-transplant anti-infective prophylaxis with acyclovir, trimethoprim/sulfamethoxazole and voriconazole/posaconazole. Blood monitoring for cytomegalovirus (CMV) reactivation using quantitative PCR (polymerase chain reaction) was performed weekly for the first 60 days and then at least once bi-weekly up to 100 days after transplantation. Cytopenia was defined according to the Common Terminology Criteria for Adverse Events, Version 4.03 [CTCAE]. All patients provided an informed consent in accordance with the Declaration of Helsinki.

### Diagnosis of SR-aGVHD

Acute GVHD was diagnosed by treating physician considering clinical, imaging and laboratory findings. The severity of aGVHD was evaluated according the Mount Sinai Acute GVHD International Consortium (MAGIC) guidelines [6]. SR-aGVHD was defined as follows: (1) progression of aGVHD within 3 to 5 days of therapy with ≥ 2 mg/kg/d methylprednisolone (MP) equivalent or (2) failure to improve with 5 to 7 days of CS treatment [7].

### Ruxolitinib dosing and definitions of response

RUX was initiated orally at 5 mg twice daily and dose was increased to 10 mg twice daily if no toxicities occurred. Response to RUX treatment was defined as follows: (1) complete response (CR)—absence of all aGVHD manifestations; (2) partial response (PR)—significant improvement (at least one grade lower) in all initially affected organs. All other types of responses were considered as treatment failures [8]. RUX-refractoriness was defined as: (1) progression of aGVHD symptoms after at least 5 to 10 days of treatment; (2) lack of improvement (at least PR) after ≥ 14 days of therapy; (3) loss of gained response at any time during treatment [9].

### Statistics

Fisher exact test was used for evaluation of the following variables and their impact on RUX efficacy: aGVHD grade, number of involved organs, cytomegalovirus (CMV) and Polyoma BKV reactivation as well as grade 3–4 cytopenia. The Kaplan–Meier was used for performing the survival curves. Testing of differences in survival curves for grouping variables was performed using the method of implementing Harrington and Fleming G-rho family functions, with weights for each decease using the log-rank test. The semi-parametric Cox regression model was used to quantify the single effects of the explanatory variables on the survival factor. Statistical calculations were performed using the R v.4.1.1 statistical computing environment (R Core Team, 2021). The significance level of the statistical tests was considered as *p* ≤ 0.05.

## Results

Eighteen patients (10 male and 8 female) were treated with RUX as a salvage therapy for GIII–IV SR-aGVHD. Median age at transplant was 43 years (range 19–68). All patients initiated RUX at 5 mg twice daily. If no limiting toxicities occurred, the dose was escalated to 10 mg twice daily (*n* = 12), the remaining 6 patients continued RUX at 5 mg twice a day. No RUX interruption was observed during therapy. Study patients received allografts for acute myeloid leukemia (AML; *n* = 8), acute lymphoblastic leukemia (ALL; *n* = 2), myelodysplastic syndromes (MDS; *n* = 2), chronic myelomonocytic leukemia (CMML; *n* = 2), primary myelofibrosis (PMF; *n* = 1), chronic lymphocytic leukemia (CLL; *n* = 1), chronic myeloid leukemia (CML; *n* = 1) and severe aplastic anemia (SAA; *n* = 1). Half of the patients were transplanted in active disease, 6 in first CR (CR1) and 3 in second or third CR (CR2/CR3). 13 patients (72%) received stem cells from HLA-fully matched donor (either related or unrelated). Three patients were transplanted from 9/10 HLA matched unrelated donors and 2 individuals received haploidentical transplantation. In total, 10 patients (56%) received myeloablative conditioning (MAC), whereas reduced-intensity conditioning (RIC) was provided for 8 (44%) subjects. Peripheral blood was a source of stem cells for all transplanted patients. Nine patients started GVHD prophylaxis with dual immunosuppression consisted of MMF + calcineurin inhibitor (either cyclosporin or tacrolimus), however, calcineurin inhibitor was discontinued shortly after transplantation in 7 individuals due to renal failure. Detailed patients characteristics are summarized in Table 1.

**Table 1:** Patient characteristics at transplant

Patients, *n*	18
Sex, male (%)	10 (56)
Age at transplant, median (range)	43 (19–68)
Disease, *n* (%)	
AML	8 (45)
ALL	2 (11)
MDS	2 (11)
CMML	2 (11)
Others	4 (22)
Disease status prior to allo-HSCT, *n* (%)	
CR1	6 (33)
CR2/CR3	3 (17)
NR	9 (50)
Source of stem cells, *n* (%)	
Peripheral blood	18 (100)
HCT-CI > 3, *n* (%)	2 (11)
Donor type, *n* (%)	
Related	4 (22)
Haploidentical	2 (11)
10/10 HLA unrelated	9 (50)
9/10 HLA unrelated	3 (17)
Donor/recipient CMV status, *n* (%)	
Positive/positive	12 (66)
Negative/negative	3 (17)
Negative/positive	3 (17)
Donor-recipient sex matching, *n* (%)	
Male to male	7 (39)
Male to female	6 (33)
Female to male	3 (17)
Female to female	2 (11)
Conditioning, *n* (%)	
Myeloablative	10 (56)
Reduced intensity	8 (44)
GVHD prophylaxis, *n* (%)	
Mycophenolate mofetil + calcineurin inhibitor*	9 (50)
Calcineurin inhibitor alone	7 (39)
PT-Cy + calcineurin inhibitor	2 (11)
Number of transplanted CD34-positive cells (× 10^6^/kg), median (range)	5.55 (3.39–9.83)
ANC > 0.5 (× 10^9^/L); median (range)	14 (11–23)
PLT > 20 (× 10^9^/L); median (range)	14 (10–26)

*ANC* absolute neutrophil count, *AML* acute myeloid leukemia, *ALL* acute lymphoblastic leukemia, *CMML* chronic myelomonocytic leukemia, *CMV* cytomegalovirus, *CR* complete remission, *GVHD* graft-versus-host disease, *MD* myelodysplastic syndromes, *NR* no response, *PLT* platelet count, *PT-Cy* post-transplant cyclophosphamide

^*^Discontinued in 7 patients

Seven patients (39%) developed GIII and 11 (61%) had GIV aGVHD. Seven patients (39%) presented multiple organ involvement. Median time from allo-HSCT to aGVHD occurrence was 36 days (range 14–147). First-line treatment consisted of MP at the maximum dose of 2 mg/kg in all patients. Second-line therapies included tacrolimus, cyclosporin, mycophenolate mofetil or ATG (see Table 2). RUX therapy was administered after median of 3 prior inefficient immunosuppressive therapies (range 2–4). Doses of steroids or other immunosuppressants were gradually reduced while patients were on RUX.

**Table 2:** Immunosuppressive treatment for GVHD prior to ruxolitinib

Patient no.	Gender, age at transplant	GVHD grade	No. of involved organs	Treatment prior to RUX	Response to RUX
#1	M, 33	III	3	MP, TAC	NR
#2	M, 43	IV	3	MP, ATG	NR
#3	F, 40	IV	2	MP	NR
#4	M, 32	IV	3	MP, ATG	NR
#5	F, 48	III	3	MP, TAC, ATG	NR
#6	M, 19	IV	2	MP, TAC, ATG	NR
#7	F, 32	III	1	MP	CR
#8	F, 68	IV	2	MP, CsA, ATG	NR
#9	M, 37	III	3	MP, TAC	PR
#10	M, 43	III	2	MP, CsA, ATG	CR
#11	M, 58	IV	2	MP, CsA, ATG	NR
#12	F, 32	III	2	MP, TAC, MMF	NR
#13	F, 45	III	2	MP, TAC, ATG	NR
#14	M, 60	IV	2	MP, TAC	NR
#15	M, 44	IV	2	MP, ATG	CR
#16	F, 43	IV	3	MP, MMF	CR
#17	F, 58	IV	2	MP, MMF, ATG	NR
#18	M, 60	IV	3	MP, TAC	NR

*CR* complete response, *GVHD* graft-versus-host disease, *F* female, *RUX* ruxolitinib, *MP* methylprednisolone, *ATG* anti-thymocyte globulin, *CsA* cyclosporine A, *M* male, *MMF* mycophenolate mofetil, *NR* no response, *PR* partial response, *RUX* ruxolitinib, *TAC* tacrolimus

The median duration of RUX treatment was 28 days (range 7–129). The overall response rate (ORR) was 28% (5/18), including 4 patients with CR and 1 with PR. Median duration of response was 83 days (range 14–682).

No significant correlation between GVHD grading and RUX efficacy was demonstrated (*p* = 0.326). Number of involved organs as well as type of affected organ also did not affect the response to RUX therapy (*p* = 0.359). Among RUX-responders, 3 patients are alive and one of them experienced chronic GVHD. Two other RUX-responders died from severe bleeding (see below).

Median follow-up from the occurrence of aGVHD was 55 days (range 29–706). At the last visit, 14 (78%) patients died. The main causes of death included progression of aGVHD with hepatorenal syndrome (*n* = 11), severe intracranial and gastrointestinal bleedings (*n* = 2): one patient died from septic shock (*Streptococcus hemolyticus*). Details are shown in Table 3.

**Table 3:** Post-transplant outcome in ruxolitinib-treated patients

Acute GVHD, *n* (%)	
Grade III	7 (39)
Grade IV	11 (61)
Organs involved, *n* (%)	
Skin	14 (78)
Intestines	17 (94)
Liver	12 (67)
Interval between transplant and onset of acute GVHD, days; median (range)	36 (14–147)
Interval between acute GVHD and RUX therapy, days; median (range)	14 (4–22)
Prior GVHD therapies, median (range)	3 (2–4)
Duration of RUX treatment, days; median (range)	28 (7–129)
Overall response, *n* (%)	
CR	4 (23)
PR	1 (5)
NR	13 (72)
Alive at last contact, *n* (%)	4 (22)
Causes of death, *n* (%)	
Progression of acute GVHD	11 (79)
Severe bleeding	2 (14)
Bacterial infection	1 (7)
Median follow-up from transplantation, days; median (range)	92 (66–853)
Median follow-up from acute GvHD onset, days; median (range)	55 (29–706)

*CR* complete response, *GVHD* graft-versus-host disease, *PR* partial response, *RUX* ruxolitinib, *NR* no response

**Fig. 1:**
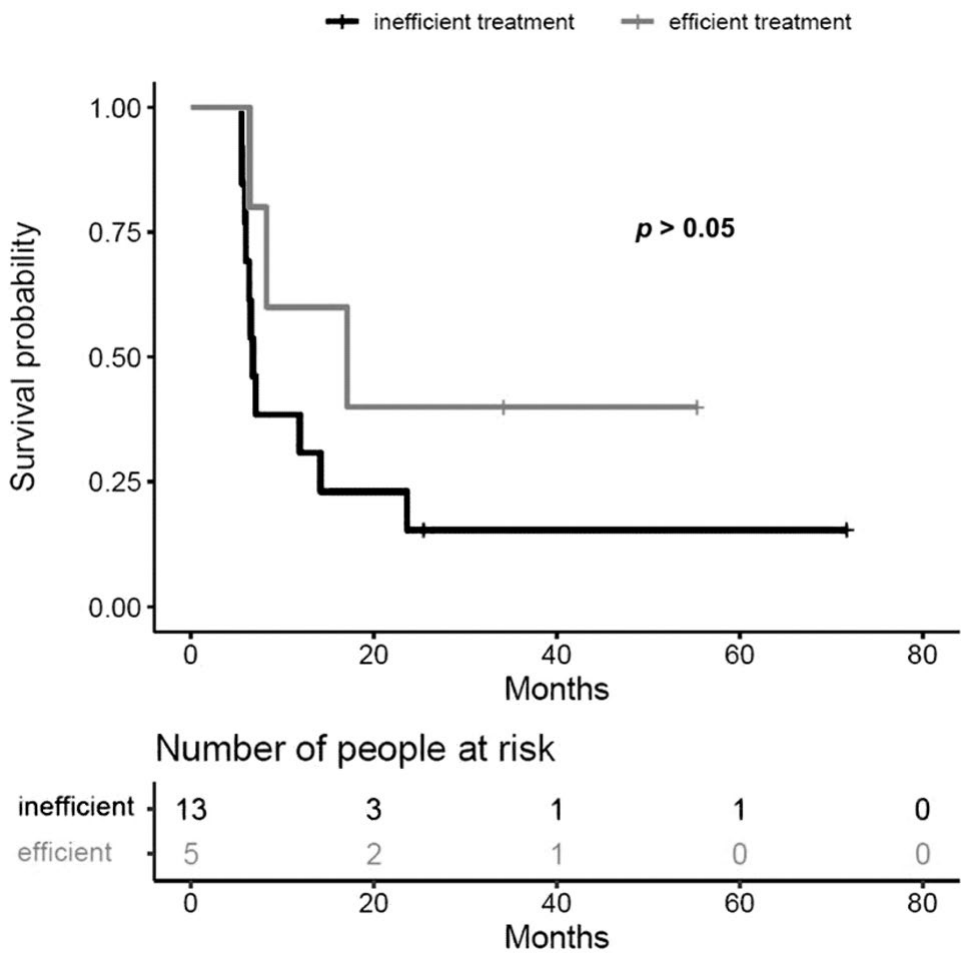
Survival curves for RUX-responders and RUX non-responders

A 12-month overall survival (OS) for RUX-responders *vs* RUX-non-responders was 60% (95% CI 29–100%) vs 31% (95% CI 14–70%); *p* = 0.200 (see Fig. 1). On univariable analysis the only factor positively influencing OS was the duration of RUX therapy above 29.5 days. The duration of treatment > 29.5 days reduced the hazard ratio by 84.6% [HR 0.154; 95% CI 0.04–0.6]; *p* = 0.007.

In general, severe cytopenias (grades 3 to 4) were the major adverse event after the initiation of RUX therapy that occurred in 89% of patients. Thrombocytopenia (*n* = 11; 61%) and neutropenia (*n* = 4; 22%) were common. Severe anemia was demonstrated in only one patient. No significant correlation between cytopenia and RUX efficacy was found. Nine patients developed CMV reactivation, polyoma BKV reactivation was demonstrated in 6 individuals and 2 patients suffered from life-threatening bacteriemia (*Streptococcus haemolyticus* and *Klebsiella pneumoniae*). The occurrence of CMV and BKV reactivation did not correlate with RUX efficacy (*p* = 0.294 for CMV and *p* = 0.294 for BKV).

## Discussion

Treatment of SR-aGVHD has remained unsatisfactory for decades. Novel therapeutic agents, which decrease aGVHD severity but preserve graft-versus-leukemia (GvL) effect and possess favourable toxicity profile remain unmet need. Janus Kinase 1 (JAK1) and 2 (JAK2) are among the most intensively studied targets due to their role in cytokine production (e.g. interleukin-1 and -6, tumor necrosis factor, interferon-gamma) and inflammatory T-cell actions [10–12]. RUX is reported to block dendritic cell activation, reduce the migration of neutrophils into GVHD affected organs and limit T-cell proliferation [13, 14].

Benefits of RUX therapy in SR-aGVHD have been showed in 2 pivotal clinical trials: REACH1 and REACH2 [5, 15]. The former focused on the efficacy of RUX treatment in combination with CS in GII–IV aGVHD. The best overall response rate (ORR) among 71 treated patients was 73% with median duration of response of 345 days. Responses were demonstrated across all affected organs, but skin remained the most prone to RUX therapy. REACH2 trial aimed to compare the efficacy of RUX *versus* standard immunosuppressive treatment. Although OS did not differ between groups, patients treated with RUX showed significantly higher ORR at day 28 (62% *vs* 39%). It should be mentioned that among studied patients, 34% manifested G II disease in which prognosis was found to be much better compared to more advanced stages. This particular group was proved to have the response rate of 75% and this was significantly higher if compared to grade III—56% and IV—53% [5].

Other studies seem to confirm the beneficial effect of RUX in SR-aGVHD. 19 patients with GIII and IV SR-aGVHD were analysed by Abedin et al. The authors reported unexpectedly promising response rate of 84% at day 28 with 6-month OS of 58% [16]. Among RUX-responders, 9 patients achieved CR and 7 patients PR. However, if compare to our study, RUX was administered as early as after median of 2 prior unsuccessful GVHD therapies. What is more, 61% of our patients had GIV disease if compared to only 21% in abovementioned study. Similarly, another analysis on 23 Chinese patients with SR-aGVHD reported almost 87% ORR for RUX treatment with 1-year OS of 82% [17]. These results were significantly better for RUX-responders than non-responders (90% vs 33.3%). Again, it should be noticed that this study included also patients with GII aGVHD (which accounts for ~ 40% of the entire cohort) and in none of the patients more than 2 organs were involved. Of note is that RUX was initiated after median of 5 days of GVHD duration.

Yet, one more real-life data reported 6-month OS of 47% for 23 patients with GII–IV SR-aGVHD (62% for RUX-responders and 28% for non-responders) [18]. Of note is that only 5 out of 23 patients reached CR during RUX. These results are in line with those presented by us.

Based on the mentioned data, one may speculate that it would be reasonable to use RUX as GVHD prophylaxis. Significantly lower incidence of GII–IV aGVHD was demonstrated for patients who received RUX as prophylaxis in comparison to other immunosuppressive drugs (42% *vs* 12.2%) [19].

Of note is that our ORR of 28% (5/18) was markedly lower than reported in REACH trials as well as in the other cited papers [16, 17]. It should be, however, mentioned that we focused only on patients with GIII–IV SR-aGVHD which is known to have the poorest outcome. From the clinical point of view this is the most demanding group of patients with the highest need for efficient salvage therapy. Furthermore, lower ORR demonstrated in our report could be partially explained by relatively late RUX initiation, namely after failure of 3 prior immunosuppressive therapies. In the RUX registration trials, RUX was administrated simultaneously with CS (REACH1) or as a second-line treatment (REACH2).

It should be mentioned that our study included relatively small number of patients and it may have an impact on the results which should be treated with caution.

Although we did not observe significant differences in mortality between RUX-responders and non-responders group, a 12-month OS stands in favour for RUX-responders vs RUX non-responders (60% vs 31%). Among all studied factors, only duration of RUX therapy over 29.5 days significantly influenced OS. No other factors showed an impact on OS in our analysis. Despite some investigations showing efficacy of RUX across affected organs [15, 18, 20], some other data bring the opposite results. None of 4 patients with SR-aGVHD and active gut involvement responded to therapy with RUX in a small study published by Neumann et al. [21]. Another report suggests that hepatic aGVHD responds worse than skin and intestines: 30% vs 79% and 71%, respectively [20]. No correlations between the type of involved organ nor the number of affected organs and RUX efficiency were demonstrated in our study.

RUX therapy is known to be associated with some side effects. Hematologic toxicity of RUX results from the blockade of JAK2 signalling processes involving thrombo- and erythropoiesis [22]. Real-life data agreeably showed high incidence of G3-4 cytopenia in 30%–60% of patients during RUX treatment [16, 20, 23]. Cytopenias were the most common adverse events also in our study. G3-4 thrombocytopenia and neutropenia were observed in 61% and 22%, respectively. Hematologic toxicity often leads to dose reductions and occasionally discontinuation of therapy, limiting the efficiency of RUX. Itacitinib- a selective JAK1 inhibitor is currently in a phase of experimental studies but some early data show that G3 cytopenia could be reduced to less than 30% [24, 25].

Among infectious complications CMV and Polyoma BKV reactivations were common: 50% and 33%, respectively. The incidence of CMV reactivation seems to be comparable with other studies. [20, 23, 26]. Till today, no data on the incidence of Polyoma BKV reactivation while on RUX were provided. Nevertheless, all these viral reactivations were manageable and did not result in patient’s death. The incidence of life-threatening bacterial infections was rare. Serious bacterial infections were demonstrated in only 2 patients with 1 death due to septic shock. In contrast, 52% of patients treated with RUX presented bacterial infection in Abedin study [16].

In conclusion, our study seems to prove that RUX could be a promising therapeutic option for SR-aGVHD, however, more data are warranted.
